# Energy-Efficient Ultrasonic Water Level Detection System with Dual-Target Monitoring

**DOI:** 10.3390/s21062241

**Published:** 2021-03-23

**Authors:** Sanggoo Kang, Dafnik Saril Kumar David, Muil Yang, Yin Chao Yu, Suyun Ham

**Affiliations:** Department of Civil Engineering, The University of Texas at Arlington, Arlington, TX 76019, USA; sanggoo.kang@mavs.uta.edu (S.K.); dafniksarilkuma.david@mavs.uta.edu (D.S.K.D.); muil.yang@mavs.uta.edu (M.Y.); yinchao.wu@mavs.uta.edu (Y.C.Y.)

**Keywords:** ultrasonic water level detection, dual microcontroller, dual targeting, cloud-based computing platform, water level changing rate, renewable energy

## Abstract

This study presents a developed ultrasonic water level detection (UWLD) system with an energy-efficient design and dual-target monitoring. The water level monitoring system with a non-contact sensor is one of the suitable methods since it is not directly exposed to water. In addition, a web-based monitoring system using a cloud computing platform is a well-known technique to provide real-time water level monitoring. However, the long-term stable operation of remotely communicating units is an issue for real-time water level monitoring. Therefore, this paper proposes a UWLD unit using a low-power consumption design for renewable energy harvesting (e.g., solar) by controlling the unit with dual microcontrollers (MCUs) to improve the energy efficiency of the system. In addition, dual targeting to the pavement and streamside is uniquely designed to monitor both the urban inundation and stream overflow. The real-time water level monitoring data obtained from the proposed UWLD system is analyzed with water level changing rate (WLCR) and water level index. The quantified WLCR and water level index with various sampling rates present a different sensitivity to heavy rain.

## 1. Introduction

Floods are one of the most frequent natural disasters, not only causing costly damage to infrastructure and property but also causing injuries and fatalities. Due to the severity of these effects, water level monitoring and flood warning are extremely important. Heavy rainfall can lead to overflow of streams and urban inundation. In some cases, flash floods that cannot be drained in time cause urban inundation without stream overflow. Thus, the monitoring of both urban inundation and stream overflow is significant to prevent losses. The water level change is one of the most common and straightforward indicators considering different channel/stream conditions such as geometrical impact, drain condition, and groundwater recharging condition. For flood monitoring, on-site water level measurement facilities are used, such as rainfall observation stations, water level observation stations, and meteorological centers. Although these on-site stations can measure the water level change and issue flood warnings instantly, it is costly to build, maintain, and operate such facilities and they pose a spatial limitation problem [1].

As the demand for accurate flood monitoring has increased, a cost-effective water level detection and real-time monitoring system has been studied in meteorology and engineering. To monitor water level, four types of water level sensors are commonly used: bubble gauges [2], float gauges [3], pressure sensors [4], and non-contact distance sensors [1,4,5,6]. Methods using the bubble gauge, float gauge, and pressure sensor are applied for a longer time than the method utilizing the non-contact distance sensor, such as ultrasound sensor [7] and radar [8]. Those devices are hard to maintain when the proper installation points are limited with additional efforts to measure the level on the site and frequent maintenance [1]. The non-contact ultrasonic sensor has been developed and deployed in many areas for those challenges [9,10]. Water level detection with non-contact sensors has been studied due to its convenience by not being directly exposed to water, which can cause constant degradation [9]. The reliability of water level measurements is critical for monitoring-based flood warnings under the changing external environment condition without false alarm. The airborne wave speed change by the temperature and moving average fitting are also considered to obtain a reliable water level with a non-contact ultrasonic sensor [7].

Real-time monitoring systems have been studied with the development of the interconnection in the network of devices. The development leads to networking capabilities between sensors and software or computing platforms, which allow users to access data in a short time [11,12,13,14]. The components of the typical monitoring system consist of a module controller, a wireless network module, a data server, and a user interface platform. The general data flow between the components is (1) the microcontroller (MCU) controls sensors, and the sensors detect the water level [4,9,10,11]; (2) the collected water level data is transmitted via the networking system, such as a wireless sensor network (WSN) [15] or a cellular network [4,9,12]; (3) the transferred data is stored to the data server, such as a web server [9,12,13,14] or a personal computer-based server [15]; and (4) the stored data is processed and presented by the computing platform, such as a web-GIS platform [11,16], customized software [15], or cloud computing platform [17].

Power management and power consumption minimization of the unit with renewable energy harvesting are also important in order to ensure a long-term stable operation. Single MCU application for the unit operating remotely at certain intervals provides a sleep mode for power saving in the MCU [4,5,9,14,18,19]; however, there is a possibility of unexpected current consumption in the single MCU system during the sleep mode from connected peripherals (e.g., sensor and other modules). Although there are efforts to utilize a self-powered water level sensor to harvest energy from liquid-solid contact [20,21,22], it is inapplicable to non-contact sensors. Advanced power management technique in programming level is studied using power management application programming interfaces (APIs) [23]. Although the advanced power management system of API method [23] and integrated circuits using Maximum Power Point Tracker chip [24] method are considerable to manage the system power, these approaches are possibly limited to specific data processing or control design.

This paper presents works conducted to overcome key technical barriers for operating a remotely controlled water level detection unit in terms of low-cost and power-efficient ultrasonic water level detection (UWLD). Furthermore, we apply a dual-targeting system, which is the first study to monitor the water level on both pavement and streamside for the urban inundation and stream overflow monitoring. For this purpose, we develop and introduce a low-cost UWLD unit using a single-MCU system and cloud computing platform. The proposed UWLD unit is evaluated in terms of power consumption and improved energy efficiency by adopting a dual-MCU system: the dual-MCU system deploys a secondary low power consumption MCU to further reduce battery consumption (see Section 2.3 for more detail). Finally, we obtain 5-month water level monitoring data from the developed UWLD system. The obtained data is analyzed with water level changing rate (WLCR) and water level index (WLI) to discuss the time-sensitive water level detection for the flood warning system. The findings reveal the potential for water level monitor presenting water level changes in higher sampling rate on both pavement and streamside and demonstrates the energy efficiency of the developed unit.

## 2. Methodology

Three versions of the low-cost system will be introduced and compared: proposed basic UWLD system (UWLD-1) with single MCU and single target; dual-target UWLD (UWLD-2) of the unit to detect the water level change on both pavement and streamside; and dual-target UWLD with highly energy-efficient design by using dual MCUs (UWLD-3).

### 2.1. UWLD-1: Development of Basic Low-Cost UWLD System

#### 2.1.1. Proposed Basic UWLD System

**Figure 1:**
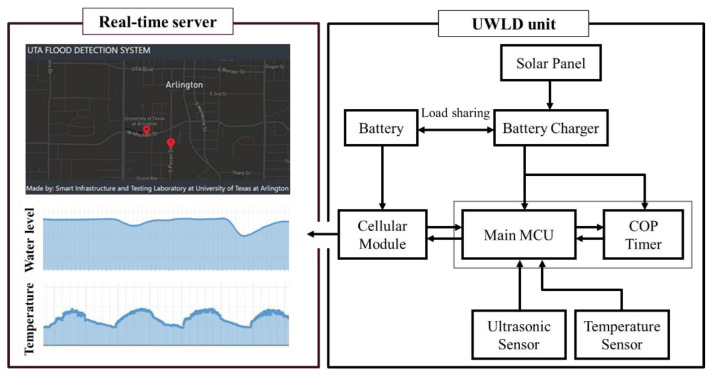
System representation of the basic UWLD system: real-time user interface platform plotting real-time temperature and water level (**left**) and UWLD-1 unit representation (**right**). It implies the data from the remote UWLD unit can be monitored in real-time on the website.

The low-cost UWLD system is developed with measuring sensors, MCU, cellular module, and a battery with a charging system. The material cost of the developed UWLD unit is relatively low (less than $250) compared to other commercial stations, and it allows more units at different locations to be installed to obtain the more localized and higher resolution of monitoring data. The main part of the UWLD system consists of low-cost units, including a data acquisition system and a real-time cloud computing platform. The system representation of the UWLD is shown in Figure 1. Two kinds of sensors, an ultrasonic sensor (MB7386 HRXL, MaxBotix, Brainerd, MN, USA) and a temperature sensor (DS18B20, Adafruit, New York, NY, USA), are deployed to collect data from the site. The ultrasonic sensor is deployed to calculate the air gap distance between the sensor and the water surface by two-way travel time (TWT) distance measurement. The sensor has a central frequency of 42 kHz with a 6 Hz maximum sampling rate providing transistor-transistor logic (TTL) serial interface. The TTL serial output includes pulse width data representing the distance between the sensor and water surface. The hard target ultrasonic sensor is selected to reduce the acoustic sensitivity since the higher acoustic sensitivity sensor possibly captures more outside noise sources as a signal. The temperature sensor is deployed to increase the accuracy of the distance from the ultrasonic sensor by correcting it with the temperature effect on the speed of sound. The low-cost MCU (ATmega644PA-PU, Microchip, Chandler, AZ, USA) is deployed to control these two sensors. For the networking system, the cellular module is deployed to transfer the data collected by the UWLD unit to the cloud computing platform server for real-time data plotting. The solar panel, battery charger, and battery are installed to supply stable power to the UWLD unit. The battery charger has a load-sharing function that allows the solar panel to charge the battery and to supply power to the system simultaneously. The load-sharing charger is effectively operated during the daytime when the solar panel generates more electricity than the unit demands for operating the UWLD system.

**Figure 2:**
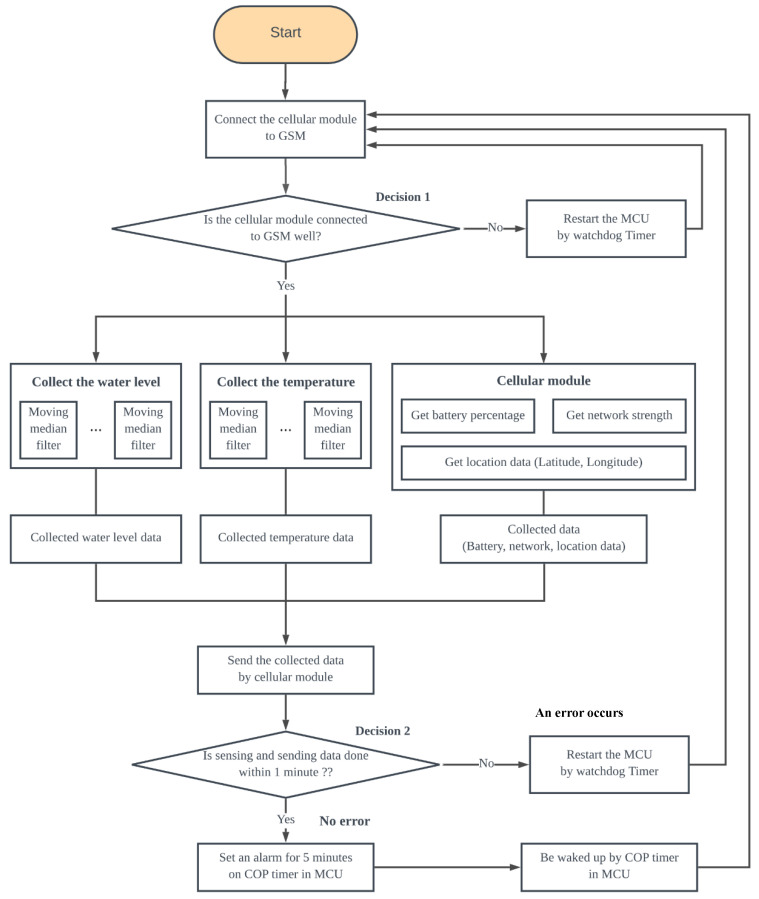
Flowchart of basic low-cost UWLD system with a single MCU and an ultrasonic sensor.

The proposed UWLD system undergoes a cycle of a process consisting of two modes: operating mode and power-saving mode. In the operating mode, the system collects water level data and sends them to the webserver. After the data collection, the UWLD system proceeds to the power-saving mode to reduce battery consumption. Sleep mode using computer operating properly (COP) timer in the MCU is initially deployed for the power-saving. This COP timer, called a watchdog timer, controls the mode of MCU by restoring the system with a certain sleep time after the operating mode. The detail of the UWLD-1 system is described in Figure 2 flowchart. The flowchart shows two decisions to prevent the system fault. Decision 1 is to check the communication between the cellular module and the MCU by inspecting the Global System for Mobile Communications (GSM) module connectivity. Decision 2 is to check the system operation by confining the completion time of one cycle of data collecting and transferring within 1 min. The single cycle is typically completed within 30 s; thus, a system fault occurs if it takes longer than a minute. With the false condition in the decision, the COP timer reboots the system to conduct the additional monitoring cycle.

#### 2.1.2. Water Level Detection

The distance measurement based on TWT of the ultrasound is influenced by the temperature. The ultrasound wave velocity in the dry air is given approximately by Equation (1) [25]:

(1) $$v_{air}\approx 331.4+0.6T_{c}\;,$$

where $v_{air}$ is wave velocity (m/s) in the air, $T_{c}$ is Celsius temperature, and 0.6 is a coefficient of the wave velocity change by the Celsius temperature. Based on the corrected wave velocity, $\;v_{air}$, the distance between the sensor and water surface can be calculated by the ultrasonic sensor as described in Equation (2).

(2) $$Distance=v_{air}\times \frac{TWT}{2}\;,$$

where *TWT* is obtained by the time of flight of ultrasonic waves between the sensor and water surface.

Although the wave velocity is corrected by the on-site temperature to achieve the reliable water level, the obtained *TWT* may provide inaccurate values due to the unexpected signal loss or energy attenuation by several factors, including the presence of objects (e.g., birds and insects) on the wave path. Therefore, the moving average data filtering [7] is not suitable in this case since averaged data include the unexpected measurements. Therefore, a moving median filter is adopted to select a reliable water level distance from the time series data. The moving median filter is the selection of the median distance among the measured distances (10 measurements by 6 Hz sampling rate of the ultrasonic sensor) arranged in ascending order as the representative distance for the measurement of the cycle.

#### 2.1.3. Cloud Computing Platform

**Figure 3:**
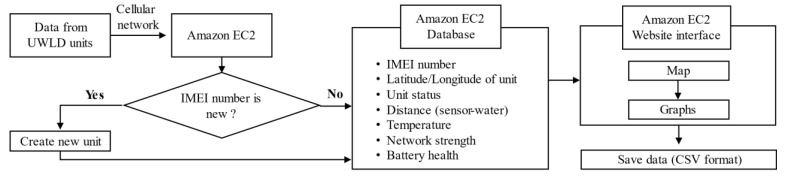
Flow chart (Programming layout) of the data processing of the UWLD system.

To monitor the water level data in real-time, an online accessible cloud computing platform is used. The website to display the transferred data from the UWLD unit and online database are created in Amazon Elastic Compute Cloud (EC2) for post-processing and saving the data. The transferred data consists of ultrasonic TWT, temperature, battery health, current network strength by the international mobile equipment identify (IMEI) number of the subscriber identification module (SIM) card. The locations of the unit are marked on the map, and the more detailed information of the node comes up when the red mark is clicked (see Figure 1 (left top)). The flow chart of the data processing of the UWLD system is described in Figure 3. For the optimized process, multiple program languages were used: MySQL for database, HTML for the website, and JavaScript for the map and graph. Thus, an application programming interface (API) allows to communicate for each different program language during processing, as interface/communicator in EC2 server. The data set can be saved in the CSV file format.

### 2.2. UWLD-2: Dual Target Sensing of UWLD

**Figure 4:**
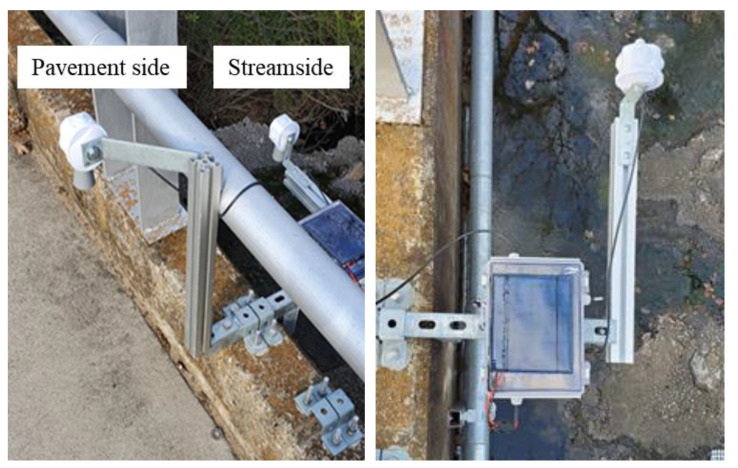
Installation of UWLD-2 system to monitor the water level on (**left**) the pavement side and streamside and (**right**) the streamside.

**Figure 5:**
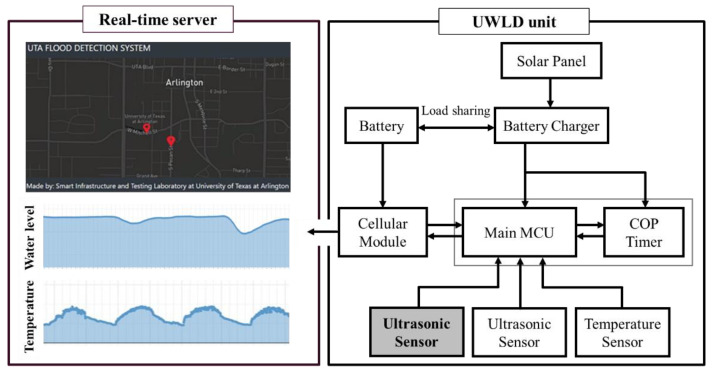
System representation of UWLD-2 with dual-target sensing. The additional ultrasonic sensor is deployed for monitoring the pavement side water level. The moving median filtered distance data from the pavement side sensor is also transferred to the cloud server.

The ultrasonic sensor is installed to calculate the distance between the sensor and the water surface on the streamside in common practice. However, if the infrastructure (e.g., pavement and bridge) cannot drain the water in time, it may cause urban inundation regardless of stream overflow. Thus, it is imperative to monitor the water level on both the pavement side and the streamside. Two ultrasonic sensors are deployed for the UWLD system (UWLD-2), as shown in Figure 4. The system representation of UWLD-2 is described in Figure 5.

### 2.3. UWLD-3: UWLD System with Duazl MCUs and Dual-Targeting

**Figure 6:**
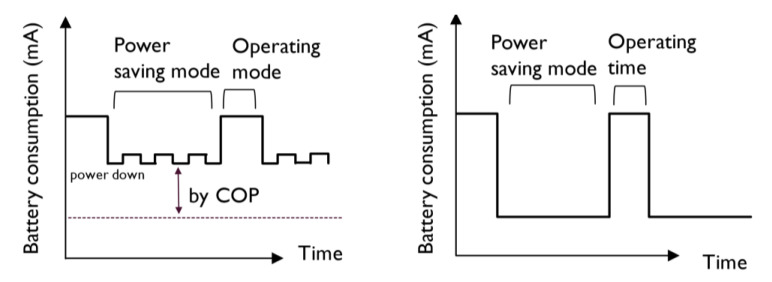
Battery consumption of operating mode and power-saving mode. The UWLD system with sleep mode by the COP timer (**left**) and an ideal power-saving mode (**right**).

The UWLD-1 and UWLD-2 units are operated under operating mode and power-saving mode by the single MCU as described in Section 2.1.1 by adopting the sleep mode function in the COP timer. In spite of operating COP timer sleep mode, the power-saving mode may provide an inefficient power operation. Due to the nature of the COP timer, the power consumption of the UWLD system possibly presents higher battery consumption periodically in the power-saving mode, as illustrated in Figure 6 (left). Theoretically, the low-power stage in the power-saving mode by COP timer presents still significant energy consumption keeping the COP timer on. To reduce the total battery consumption, the ideal power-saving mode should have minimum battery consumption during the power-saving mode, as shown in the conceptual plot in Figure 6 (right).

**Figure 7:**
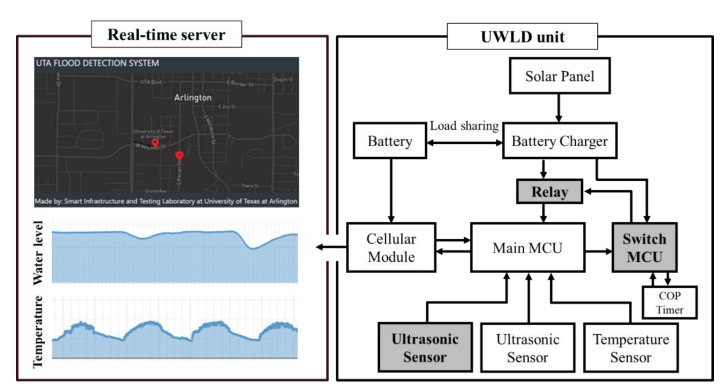
System representation of UWLD-3 system with dual-target sensing and dual MCUs.

To further reduce battery consumption, a dual MCU system is designed toward a simple and broad application for a remote sensing unit, avoiding complex data flow and programming but providing considerable power savings. The system representation with the UWLD unit, including the dual-MCU system, is described in Figure 7. Dual-MCU system comprises the main MCU, an additional MCU (ATtiny25-20PU, Microchip, Chandler, AZ, USA) which has low power consumption, and a solid-state relay (AQY282EH, Panasonic, Osaka, Japan) module are installed in between the battery charger and the existing MCU. The main MCU is the high-performance MCU to perform the main task, including collecting and sending data. The additional low-power MCU, which is called as a switch MCU, is then installed for turning on and off the main MCU with a solid-state relay (SSR) that normally consumes less power than electromagnetic relay. Although two MCUs consume the same amount of power (0.1 µA at 1.8V) in the sleep mode, the main MCU presents unexpected high current consumption during the sleep mode from peripheral sensors and devices that are directly connected to the main MCU. Shutting down completely the high energy-consuming main MCU during the power-saving mode keeps the system in minimum power consumption, although the switch MCU consumes power in the power-saving mode by COP timer. Thus, the primary purpose of the switch MCU and SSR is to shut down the main MCU and other devices. The dual-MCU system maintains the power-saving mode with minimum power consumption by controlling the main MCU power supply. The switch MCU sends a signal to the relay module to turn off the main MCU after one cycle of the operation, and it goes to the sleep mode by COP timer. At the next cycle, the COP timer turns on the switch MCU, and the switch MCU sends a signal to the relay module for restoring the main MCU process.

**Figure 8:**
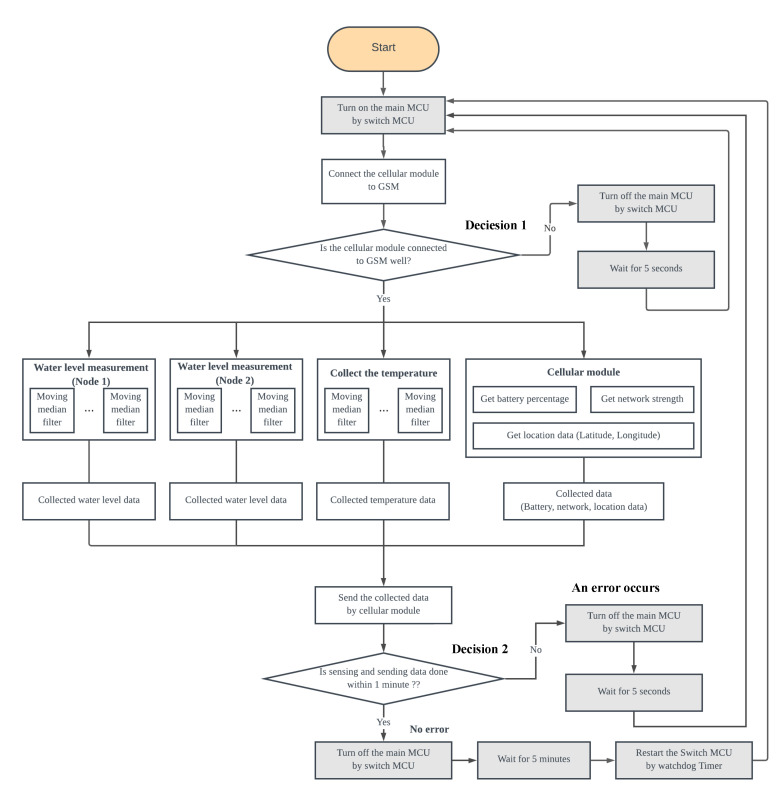
Flowchart of the UWLD system with dual-target sensing and dual MCUs (UWLD-3).

The power efficiency of UWLD-1 and UWLD-3 is discussed in Section 3.2. The detailed flowchart is shown in Figure 8, with a highlighted dual-MCU system. The dual-MCU system is also designed to have a 5-min data sampling rate for the operating mode cycles. The communication between the main MCU and switch MCU is described in the flowchart with the two decisions described in Section 2.1.1.

## 3. Results and Discussion

### 3.1. Unit Installation and Calibration

**Figure 9:**
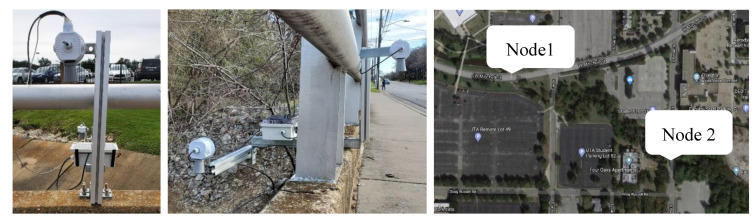
Installed units and locations: Node 1 (**left**), Node 2 (**middle**), and location map of two nodes (**right**).

Two UWLD units are installed on West Michell St. bridge and South Pecan St. bridge (Arlington, Texas, USA), which have a 0.3 km distance (see Node 1 and Node 2 in Figure 9 (right)). Initially, UWLD-1 and UWLD-3 units are tested for the comparative study of energy efficiency. Ultimately, the UWLD-3 unit is adapted to monitor the water level changes for 5 months from March to July 2020 for each node. The installed units and location are shown in Figure 9. Each node has two ultrasonic sensors to measure for both the pavement side and streamside, and the solar panel is installed on the top of the control box at the streamside.

**Figure 10:**
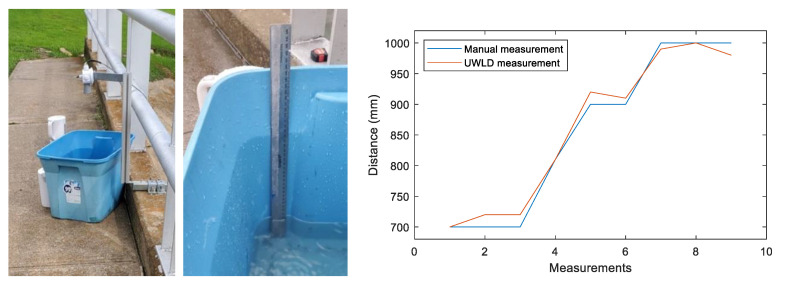
Ultrasonic sensor calibration test (**left**) and measured data (**right**).

The calibration test is conducted to study the accuracy of water level detection by comparing the distances from the ultrasonic sensor measurement and the manual measurement, as shown in Figure 10. The comparison between the manually measured distance and the ultrasonic sensor distance is shown in Figure 10 (right). The blue line in the graph indicates the manually measured water depth in the bucket, and the red line indicates the water depth data obtained from the ultrasonic sensor. Four distances are tested, 700 mm, 800 mm, 900 mm, and 1000 mm. The UWLD system measures the distance in 1-mm resolution, and the calibration result shows 0 to 20 mm error. To reduce the error, the moving median filter is utilized as described in Section 2.1.2.

### 3.2. The Energy Efficiency of the UWLD Unit

**Figure 11:**
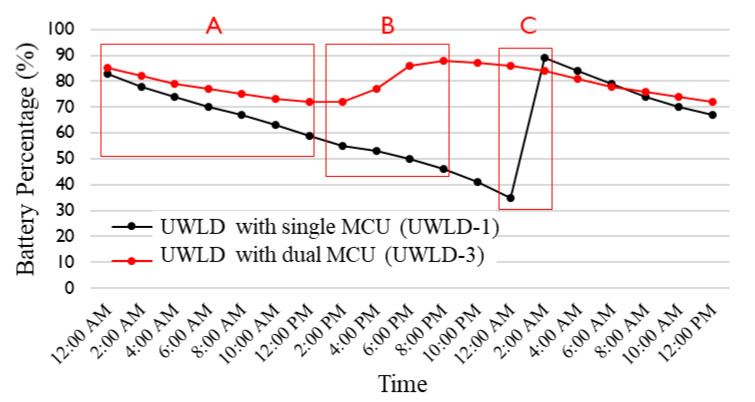
Thirty-six h monitoring of battery percentage record of the UWLD system with the single-MCU system (UWLD-1) in black and dual-MCU system (UWLD-3) in red under similar sunlight conditions. A, B, and C regions indicate battery consumption at the nighttime and daytime and battery replacement time (battery replacement is for only UWLD-1 unit). The result implies the dual-MCU system improves the energy efficiency of the UWLD unit without changing the battery for longer operation period.

Both stabilizing the power supply from the solar panel with battery and minimizing the unit’s power consumption are significant to maintain the reliable UWLD system. Figure 11 shows the remaining battery percentage of the single-MCU unit (UWLD-1) and dual-MCU unit (UWLD-3) for 36 h on both similar sunlight conditions. For consistent battery consumption test, testing dates are selected under similar environmental condition; the duration between the sunrise and sunset is 11 h, 10 min for a single MCU and 11 h, 9 min for dual MCU; the average percentage in each cloud cover is 49.9% for single MCU and 50% for dual MCU. Although the result might be altered by the recharge of the batteries tested, each shortwave solar power at the region is the same as 0.63kW. Region A and B indicate the nighttime and daytime battery consumption, respectively. C region indicates the time we replaced the battery for re-operating the UWLD-1 unit. The UWLD-1 unit using the COP timer of the single MCU presents more than 2% of the battery usage per hour (48% loss per day), which makes the unit run out the power in 2 days, as shown in the A region of Figure 11 (black). The UWLD-1 unit shows improper battery charging by the solar panel in region B. The dual-MCU system introduced in Section 2.3 shows less than 1% power consumption per hour (24% loss per day), as shown in the A region in Figure 11 in red. The dual-MCU system allows proper battery charging in the B region. The comparison study implies that the dual-MCU system decreases the power consumption of the unit while UWLD-3 unit operates two ultrasonic sensors and improves the power charging efficiency.

**Figure 12:**
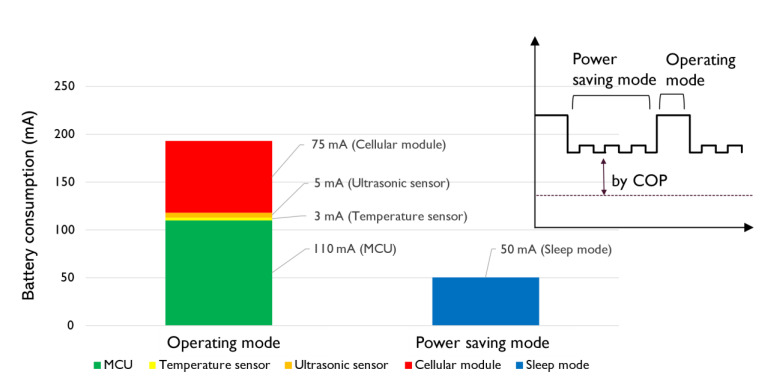
Battery consumption by components under the operating and power-saving mode in the single MCU system (UWLD-1). The results imply most of the battery power is consumed by MCU operation in both operating mode and power-saving mode.

Figure 12 shows the details of battery consumption from each UWLD-1 component, cellular module, ultrasonic sensor, temperature sensor, and MCU. Total of 185 mA and 50 mA batteries are consumed under the operating mode and the power-saving mode, respectively. The battery consumption by the components implies that most of the battery is consumed by MCU in both modes; thus, the dual-MCU system is developed to improve the energy efficiency at the MCU part.

**Figure 13:**
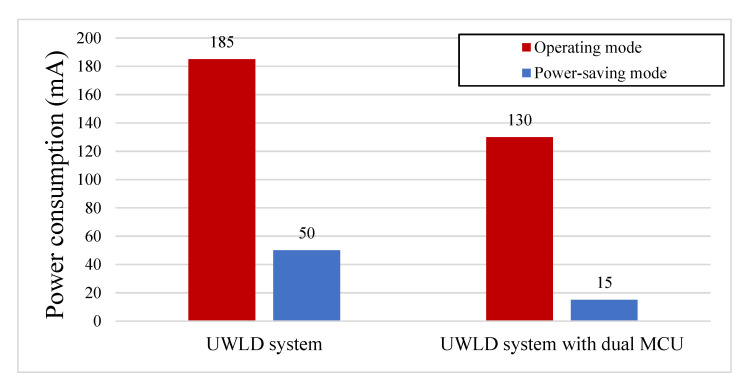
Power consumption of single-MCU and dual-MCU system, presenting the energy efficiency improvement with the dual-MCU system in both operating mode and power-saving mode. A 30% and 70% improved energy efficiency is shown under the operating mode and power-saving mode, respectively.

The battery consumption in the operating mode and power-saving mode of the single- and the dual-MCU system is described in Figure 13. Under the operating mode, the dual-MCU UWLD unit shows 30% decreased power consumption than the single MCU controlled by COP timer from 185 to 130 mA. The improved power efficiency in the operating mode of the dual-MCU system indicates the main MCU controlled by COP timer takes more power than completely turned on and off by the switch MCU. Under the power-saving mode, the energy efficiency is significantly higher by decreasing the power consumption by 70% (50 mA to 15 mA). Since the switch MCU is used not only under the power-saving mode but also in the operating mode as described in the flow chart of Figure 8, the energy efficiency is improved in both modes. This improvement leads to the reduced power consumption in the dual-MCU system as described in Figure 11.

### 3.3. Rainfall Monitoring with UWLD System

#### 3.3.1. Relation of Water Level Changes by UWLD System and NOAA

**Figure 14:**
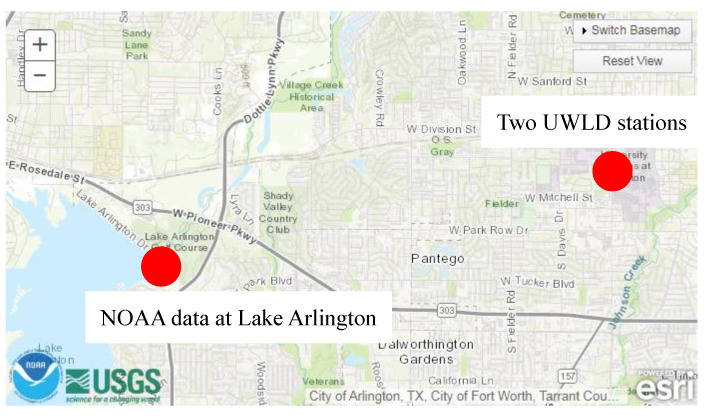
Map of the water level monitoring locations by NOAA and UWLD system. Lake Arlington is the closest gauge location, which is operated by NOAA, located in north Texas.

The water level monitoring data obtained from the National Oceanic and Atmospheric Administration (NOAA) are used to compare with the data obtained by the proposed UWLD system to understand the tendency of water level changes in different bodies of water. The location of the chosen NOAA water level monitoring node is at Lake Arlington (Arlington, Texas, USA), which is the nearest node within a 5-mile distance from the two UWLD nodes, as shown in Figure 14.

**Figure 15:**
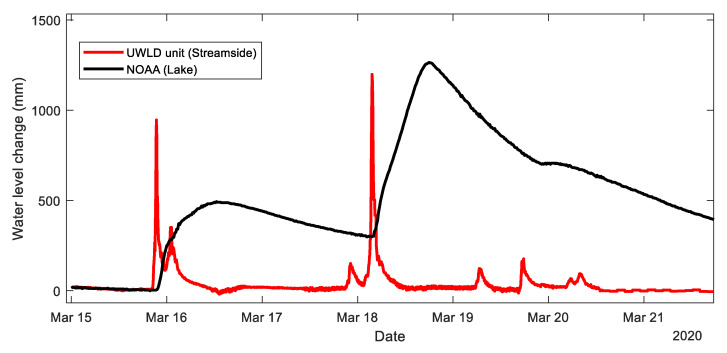
Water level changes measured by Node 1 streamside and Lake Arlington NOAA for 7 days (15–21 March 2020).

Figure 15 shows the water level changes for 7 days (15 March to 21 March) monitored by the UWLD system (red line) and NOAA data (black line), which are at the University of Texas at Arlington (see Figure 9 right map) and Lake Arlington, respectively. During the period, 8 times of water level peaks on streamside are monitored in UWLD data, while the water level data at Lake Arlington shows two relatively gradual peaks. The monitored tendency difference in Figure 15 indicates that the stream water level changes caused by heavy rainfall are more sensitive than the water level data at the lake. It implies the water level detection at the stream level, which is a relatively smaller water body, is better for detecting flash flooding, although the NOAA data is useful to observe and maintain the water resource for the larger scale. The figure shows the lake water level increases happened later than stream water level increases.

#### 3.3.2. Correlation of Node 1 and Node 2 UWLD System

**Figure 16:**
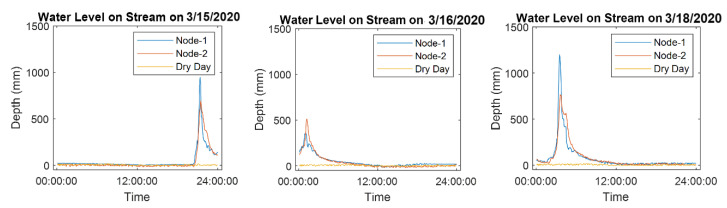
Monitored data in three rainfall events from Node 1 and 2 streamside compared with dry day’s reference water level in yellow.

The two UWLD units, Node 1 and Node 2, are installed under a similar environmental condition of a stream in 0.3 km distance. Thus, the monitoring results exhibit a similar trend of the water level changes on the streamside. The similarity of two rainfall curves by two nodes is defined by calculating the correlation coefficient [26]. Figure 16 shows the water level changes in the three different precipitation events.

The calculated correlation coefficients are shown in Table 1. The correlation coefficient is calculated by the rainfall curve at each event. The averaged correlation coefficient is 0.932. The correlation analysis indicates that the measured distance data from two UWLD units show a strong correlation, and it implies the relatively closer locations than the rainy area shows a similar tendency of water level change.

#### 3.3.3. Relation of Pavement and Streamside Water Level in UWLD-2

**Figure 17:**
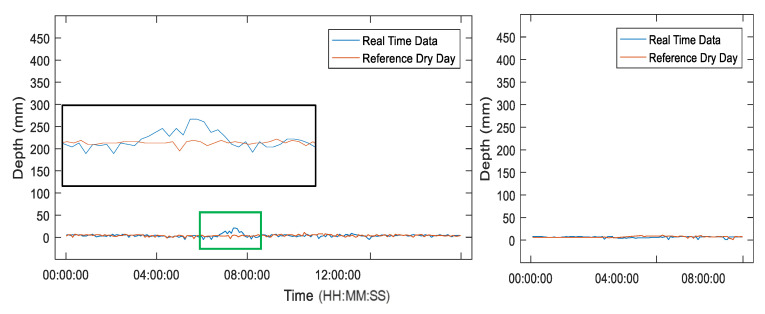
Pavement-side water level changes on 6 July Node 1 (**left**) and Node 2 (**right**).

In the periodic monitoring, the water level change on the pavement side of the bridges presents extremely small water level changes due to the good drainage system of the bridge. However, for a certain case (e.g., flash flood, heavy rainfall, drainage issue), the water level may rise quickly on the pavement side, as shown in the result of Node 1 (see Figure 17). This is possibly due to the different bridge drainage conditions, causing water stagnation. In comparison, Node 2 on the same day shows no water level changes under the good drain of water at the pavement side. Although, in one case, the pavement-side water level change is detected in the monitoring period, it is potentially significant for monitoring the urban inundation when the location is continuously exposed to the bad drainage condition.

#### 3.3.4. Analysis of the Water Level Monitoring Results

**Figure 18:**
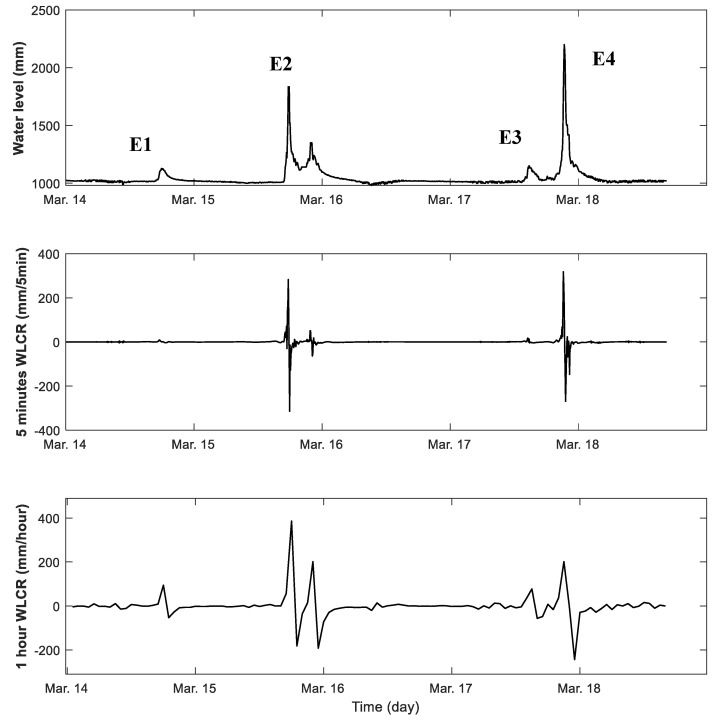
WLCR of 5 days rainfall (14–18 March): (**top**) water level change, (**middle**) WLCR by the 5-min sampling rate, and (**bottom**) WLCR by the 1-h sampling rate. The three graphs showing the WLCR by the 5-min sampling rate show the more sensitive behavior at the higher WLCR case than the 1-h sampling rate.

The water level changing rate (WLCR) adopts rainfall intensity which is one of the commonly used rainfall characteristics defined as the amount of rain falling per unit of time [27]. The WLCR is calculated as the ratio of the amount of water level change and unit time. In general, the higher sampling rate allows more sensitive detection of water level changes in flash flooding cases than the lower sampling rate. The developed UWLD system adopts the 5-min sampling rate for the water level monitor considering the energy efficiency and unit maintenance since the more frequent operation consumes more power (see operation mode in Section 3.2). The analysis of the water level monitoring results in different sampling rates is performed. The comparison between the WLCR based on the 5-min and 1-h data sampling rate is described in Figure 18. The water level record covers 5 days, 14 March to 18 March, and four rainfall events (E1–E4) are highlighted. The E1 and E3 rainfall events show a relatively moderate water level change than the E2 and E4. The 5-min WLCR curve shows more sensitive behavior at the dramatical rainfall than 1-h based WLCR curve.

**Figure 19:**
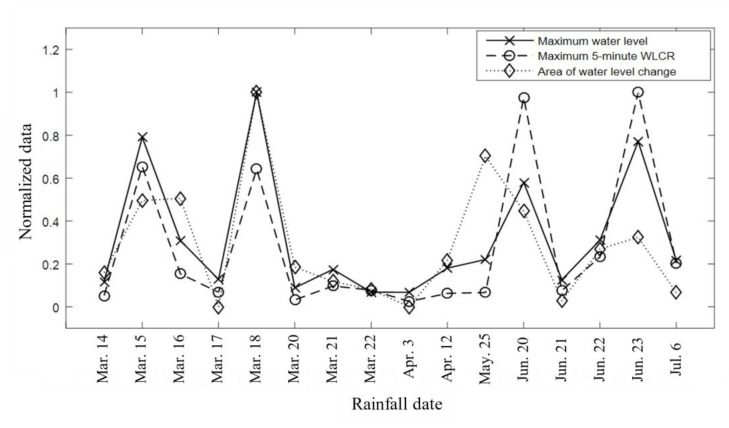
Normalized maximum water level, WLCR, and area data with the 16 rainfall events are presented. The results indicate a similar tendency by the rainfall events.

The maximum water level, WLCR, and area of the water level change of 16 rainfall events are quantified in Figure 19. The maximum water level is the peak point on the water level curve, and the quantified area is the area under the water level change curve. The correlation coefficient between maximum water levels and rainfall intensities is calculated as 0.88, which is a ‘highly linear correlation’ [26]. The correlation coefficient between maximum water levels and areas of the water level change is calculated as 0.74. The maximum water level, which is a significant indicator to monitor flash flooding, is more related to the WLCR than the area affected by the rainfall duration. The highly correlated WLCR and maximum water level relation indicates that the heavy rain in a short period of time causes the greater water level rise than the large amount of water in a long period of time rainfall.

The water level index (*WLI*) is adopted to present water level changes considering the previous changing tendency. *WLI* is calculated by the water resources index method based on rainfall accumulations over time with a weighting factor [28]. The water level data is used to calculate *WLI* instead of precipitation data which is usually collected hourly or daily in the water resources index method. The weighting factor is calculated by the number of data points considering window size to get *WLI*. For example, *WLI*_1*hr*_ is calculated by 1-h data windowing with 12 water level data points (*L*_1_*–L*_12_) which is obtained by the 5-min data sampling rate. *WLI*_1*hr*_ is expressed:

(3) $$WLI_{1hr}=L_{1}+\frac{\left\{L_{2}\left(W_{12}-1\right)\right\}}{W_{12}}+\frac{\left\{L_{3}\left(W_{12}-1-\frac{1}{2}\right)\right\}}{W_{12}}+\frac{\left\{L_{4}\left(W_{12}-1-\frac{1}{2}-\frac{1}{3}\right)\right\}}{W_{12}}\;+\ldots +\frac{\left\{L_{12}\left(W_{12}-1-\frac{1}{2}-\ldots -\frac{1}{11}\right)\right\}}{W_{12}},.\;$$

where $W_{12}$ is a weighting factor by 12 data points (1-h data windowing), and it is expressed:

(4) $$W_{12}=1+\frac{1}{2}+\frac{1}{3}+\ldots +\frac{1}{12}\;.$$

**Figure 20:**
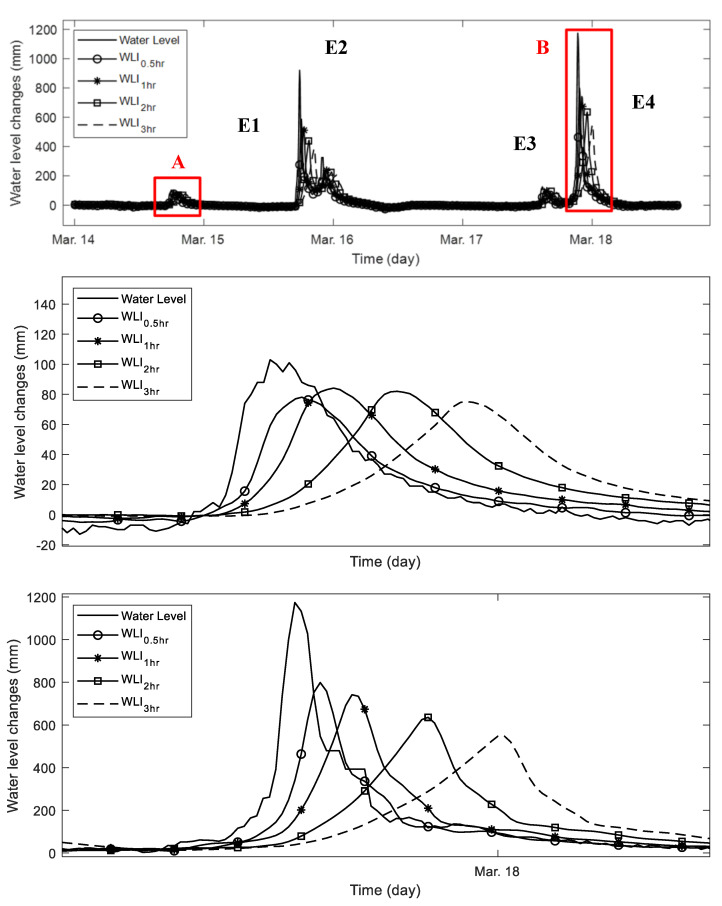
Water level and WLI changes; (**top**) water level change and 4 different WLI curves based on 0.5-, 1-, 2-, and 3-h time windows; (**middle**) the zoomed-in plot of the E1 rainfall event, region A of the top figure; and (**bottom**) zoomed-in plot of the E4 rainfall event, region B of the top figure. The WLIs calculated by different time windows show the more sensitive change in the rapid water level change case (B region) than the relatively moderate water level change case (A region).

Figure 20 shows the water level, *WLI*_0.5*hr*_, *WLI*_1*hr*_, *WLI*_2*hr*_, and *WLI*_3*hr*_ curves monitored for the same 5 days rainfall (14–18 March) in Figure 18 (top). The WLIs are calculated in four different time windows: 0.5-, 1-, 2-, and 3-h. Thus, *WLI*_0.5*hr*_, *WLI*_1*hr*_, *WLI*_2*hr*_, and *WLI*_3*hr*_ have different data points: 6, 12, 24, and 36 by the 5-min sampling rate. The weighting factors ($W_{6},\;W_{12}$, $W_{24},$ and $W_{36}$) of *WLI*_0.5*hr*_, *WLI*_1*hr*_, *WLI*_2*hr*_, and *WLI*_3*hr*_ are calculated in the same manner of Equation (4). All of the *WLI* curves near the E1 rainfall event show similar shape and peak with water level change, while the *WLI* curves near the E4 rainfall event, which is a relatively sudden change of water level, exhibit lesser correlated behavior than the first rainfall event. Therefore, *WLI* analysis with different time windows provides the rate of water level change by indicating the presence of changing shape in graph.

## 4. Conclusions

The real-time water level monitor is conducted by the developed UWLD unit using the real-time monitoring system through the cloud computing platform. A Dual-MCU system is developed to build an efficient power supply and consumption system, and dual targets of the water level detection are considered to monitor the water level change on both pavement side and streamside. The developed UWLD system and analysis of 5 months of monitoring results represent the below conclusions:

- The UWLD system is developed to measure the water level and transfer the data to the AWS server; the obtained water level is calibrated with the ultrasound wave velocity change by the temperature. In addition, the accuracy of the distance measurement is verified with manually measured distance.
- The battery power efficiency is improved for the stable operation of the UWLD system deploying the dual-MCU unit, which is composed of the additional MCU (switch MCU) and SSR for controlling the main MCU. The dual-MCU system reduces the power consumption by 30% from 185 mA to 130 mA under the operating mode and 70% from 50 mA to 15 mA under the power-saving mode. It is significant for saving power while the solar panel charges the battery which is affected by the external environment (e.g., rainfall and cloudy day). The improved power efficiency leads to the stable operation without changing the battery under the sufficient sunlight condition.
- From the UWLD system, a total of 16 events of water level change were detected in the monitoring period. When the streamside water level increases from our UWLD system, the lake water level data either increases later or does not show water level change. This result implies the water level change at a local small creek or stream is a more sensitive flood level indicator than at the large water bodies (e.g., lake or sea level) due to its low sensitivity of flash flooding. Thus, it is significant to perceive the intensity of the rainfall from multiple locations of small bodies of water.
- The water level change on the pavement side is not always detected as streamside water level change due to the good drainage system. Although the pavement-side water level changes are more affected by infrastructure conditions (e.g., the drainage system) than the direct WLCR, it can be more significant to warn of the possibility of urban flooding, especially close to the area of the UWLD node. Therefore, the water level monitoring of dual targets, pavement side and streamside, can give more reliable and sensitive information to perceive and forecast the urban flash flooding.
- WLCR and WLI can indicate the severity of water level change. Both indicators show the more sensitive behavior at the higher WLCR case than lower absolute WLCR case at a certain level of the sampling rate. It implies the real-time analysis with WLCR, and WLI can realize the flash flooding monitoring.
- Study of unit maintenance and system protection under different environments will be considered in future research, including different water flow conditions and different protection strategies of the sensors from the environmental conditions.

## Figures and Tables

**Table 1:** The correlation coefficient of streamside water level changes from two nodes on 10 different precipitation events in 2020 (averaged correlation coefficient: 0.932).

Date	3/14	3/15	3/16	3/18	3/20	3/21	4/3	4/12	5/25	6/1
Correlation coefficient	0.9257	0.9270	0.9391	0.9047	0.9325	0.9617	0.8930	0.9481	0.9501	0.9367

## Data Availability

The data presented in this study are available on request from the corresponding author.
